# Global Research Trends in Anticoagulation and Mechanical Thrombectomy for Iliofemoral Deep Vein Thrombosis: A Bibliometric Analysis (2000–2024)

**DOI:** 10.7759/cureus.86924

**Published:** 2025-06-28

**Authors:** Anas Alhur, Ghaidaa Abdulrahman S Alqahtani, Abdulelah Nasser S Alghaeb

**Affiliations:** 1 Health Informatics, University of Hail College of Public Health and Health Informatics, Hail, SAU; 2 College of Medicine, King Khalid University, Abha, SAU

**Keywords:** anticoagulation, bibliometric analysis, dimensions database, iliofemoral deep vein thrombosis, mechanical thrombectomy, vascular intervention

## Abstract

This bibliometric study provides the first comprehensive analysis of global research trends, collaborative networks, and citation metrics related to anticoagulation and mechanical thrombectomy in iliofemoral deep vein thrombosis (DVT). Using Dimensions AI, 234 articles published between 2000 and 2025 were identified through a targeted search of terms including “iliofemoral DVT,” “anticoagulation,” and “mechanical thrombectomy.” Data extracted included publication year, citation count, research category, author affiliation, country of origin, and co-authorship. The study presents an overview of recent global research activity related to anticoagulation and mechanical thrombectomy in the context of iliofemoral deep vein thrombosis (DVT). It highlights key publication trends, leading contributors, and thematic focus areas within the literature. The analysis also outlines the collaborative nature of research efforts and shifts in scientific emphasis over time. Keyword trends indicated a shift from anticoagulation to mechanical thrombectomy as a research focus. Visual mapping using VOSviewer revealed strong institutional and international collaborations and distinct co-citation clusters. These findings highlight evolving priorities in iliofemoral DVT research and provide a foundation for future studies and collaborative efforts in vascular interventions.

## Introduction and background

Iliofemoral deep vein thrombosis (DVT) is a serious form of lower-extremity venous thromboembolism that can lead to long-term complications, such as post-thrombotic syndrome (PTS), characterized by chronic leg pain, swelling, and reduced mobility. Standard treatment with anticoagulation therapy prevents further clotting but does not actively remove the existing thrombus, leaving many patients at risk for lasting venous damage [1].

Mechanical thrombectomy (MT), a minimally invasive procedure that physically removes clots using catheter-based devices, has emerged as a promising alternative, especially for patients with extensive iliofemoral involvement [2]. In some cases, MT is combined with pharmacomechanical thrombolysis, which uses clot-dissolving drugs alongside mechanical devices to restore blood flow more effectively.

Several key clinical trials have explored these approaches. The ATTRACT trial, a large randomized controlled study, evaluated pharmacomechanical thrombolysis but found no significant reduction in PTS across all DVT types. However, a subgroup analysis suggested a potential benefit for patients with iliofemoral DVT [3]. The CAVENT trial demonstrated improved venous patency and reduced PTS at two years among patients receiving catheter-directed thrombolysis for proximal DVT [4]. While these studies advanced the evidence base, their mixed results and varying protocols have led to ongoing debate over the role of MT in standard care.

Given the expanding use of thrombectomy and the rapid evolution of clinical practices, a bibliometric analysis can offer valuable insight into how global research efforts have responded. This method allows us to trace publication trends, identify leading authors and institutions, map keyword developments, and examine international collaboration. Bibliometric tools are especially useful for detecting knowledge gaps and underrepresented regions or populations in the literature.

This study aims to provide a comprehensive bibliometric analysis of research on anticoagulation and mechanical thrombectomy for iliofemoral DVT published between 2000 and 2025. The objectives are: (1) to assess temporal trends in publication volume; (2) to identify key contributors, institutions, and countries; and (3) to visualize thematic patterns and keyword evolution using co-authorship and co-citation mapping. This analysis builds a clearer picture of where the field has been and where it should go next.

## Review

Study design

This study employed a descriptive bibliometric analysis to explore global research trends related to anticoagulation and mechanical thrombectomy in iliofemoral DVT between 2000 and 2025. The goal was to assess publication patterns, identify leading contributors, and visualize keyword and collaboration networks.

Data source and search strategy

Data were retrieved from the Dimensions AI database, known for its broad coverage of biomedical and clinical literature [5]. The following Boolean search was conducted on June 5, 2025:

(“iliofemoral deep vein thrombosis” OR “iliofemoral DVT”) AND (“anticoagulation” OR “anticoagulant therapy” OR “mechanical thrombectomy”)

The search was limited to the title and abstract fields, English-language articles, and journal articles published between January 1, 2000, and December 31, 2025. This search yielded 234 articles.

Data extraction

For each included article, key bibliographic information was extracted from the Dimensions platform. These data fields included publication title, author names and affiliations, journal name, year of publication, citation count, and assigned research categories. The database’s structured classification system was used to identify thematic categories such as “Biomedical and Clinical Sciences” and “Cardiovascular Medicine.” Citation data were cross-verified for accuracy.

The dataset was exported in CSV format and managed using Microsoft Excel (Microsoft Corp., Redmond, WA, USA). Data processing and cleaning were completed prior to visualization and analysis.

Bibliometric analysis

The cleaned dataset was analyzed using RStudio (version 2024.03; Posit Software, Boston, MA) and the Bibliometrix package [6]. Descriptive analyses included the annual number of publications, total citation counts, and institutional contributions. Citation metrics, such as total citations per article and average citations per year, were calculated. Additionally, keyword co-occurrence mapping and author collaboration networks were generated using VOSviewer (version 1.6.20) [7]. This allowed the identification of high-frequency terms and the visualization of thematic clusters.

Quality assurance and reproducibility

To ensure reproducibility, the entire workflow, from search execution to final visualization, was independently replicated by a second reviewer. All exclusions and adjustments were logged. The methodological approach was designed to be replicable using the same search query and filters applied in Dimensions AI.

Ethical considerations

As this study relied exclusively on publicly accessible metadata and did not involve human participants, patient records, or confidential information, it was exempt from institutional ethical review.

Results

A total of 234 peer-reviewed articles on iliofemoral DVT involving anticoagulation and/or mechanical thrombectomy were published between 2000 and 2025. As shown in Table 1, publication activity was modest in the early 2000s, with notable growth beginning in 2010 and peaking at 26 articles in 2024. A slight decline followed in 2025 (n = 9), reflecting typical end-of-cycle indexing delays or saturation of recent trial data.

**Table 1:** Annual number of publications (2000–2025)

Year	Publications	Year	Publications
2000	2	2013	4
2001	2	2014	9
2002	3	2015	9
2003	4	2016	7
2004	4	2017	11
2005	4	2018	8
2006	4	2019	14
2007	4	2020	16
2008	5	2021	21
2009	6	2022	17
2010	6	2023	15
2011	7	2024	26
2012	8	2025	9

The articles received over 5,000 citations, with a mean of 22.09 citations per article, indicating sustained academic interest. Highly cited studies included large-scale comparative trials of pharmacomechanical thrombectomy and anticoagulation, as well as systematic reviews that informed evolving clinical guidelines.

Using Dimensions AI's classification system, the majority of studies were categorized under Biomedical and Clinical Sciences (n = 229) and Clinical Sciences (n = 158), followed by Cardiovascular Medicine and Haematology (n = 64). Smaller thematic groups included Reproductive Medicine and Health Sciences (Table 2), emphasizing the predominance of vascular and procedural focus areas within the literature.

**Table 2:** Top research categories

Research Category	Publications
Biomedical and Clinical Sciences	229
Clinical Sciences	158
Cardiovascular Medicine and Haematology	64
Reproductive Medicine	20
Health Sciences	12

Authorship analysis identified several leading contributors. Suresh Vedantham, Anthony J. Comerota, and Stephen Alan Black were among the most prolific authors, each with 11-12 publications. Per Morten Sandset and Carl-Erik Slagsvold also contributed substantially (Table 3). The most productive institutions included Washington University in St. Louis, Inova Alexandria Hospital, and Oslo University Hospital.

**Table 3:** Most prolific authors and institutions

Author	Affiliation	Publications
Suresh Vedantham	Washington University, USA	12
Anthony Comerota	Inova Alexandria Hospital, USA	12
Stephen Alan Black	St Thomas' Hospital, United Kingdom	11
Per Morten Sandset	University of Oslo, Norway	10
Carl-Erik Slagsvold	Oslo University Hospital, Norway	10

**Figure 1:**
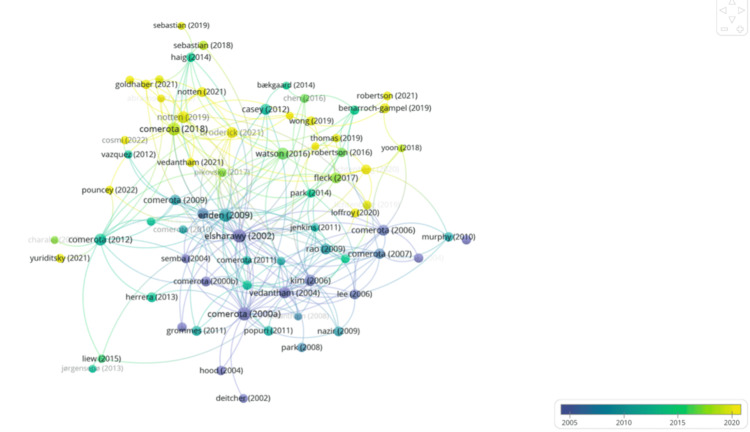
Author collaboration overlay map

A co-authorship overlay visualization (Figure 1) demonstrated dense collaborative structures, with central roles held by Vedantham, Comerota, and Black. The color gradient shows how author participation has evolved from foundational work in the early 2000s (blue) to more recent active clusters (yellow). This reflects both the continuity and renewal of collaborative networks over time.

**Figure 2:**
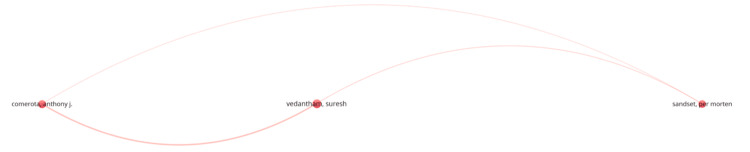
Focused co-authorship network of leading researchers in iliofemoral DVT research DVT: deep vein thrombosis

A focused author-level collaboration network (Figure 2) further highlighted a triadic partnership between Vedantham, Comerota, and Sandset, underscoring their leadership in multicenter trials and translational research in thrombosis management.

**Figure 3:**
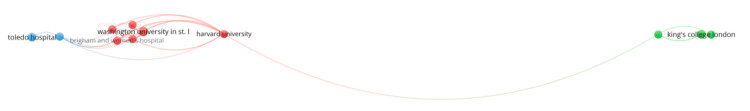
Institutional co-authorship network in iliofemoral DVT publications DVT: deep vein thrombosis

At the institutional level, co-authorship mapping (Figure 3) revealed prominent U.S.-based research clusters. Washington University, Brigham and Women’s Hospital, and Harvard University showed strong inter-institutional links. Notably, King’s College London and Toledo Hospital formed transatlantic ties, demonstrating emerging international engagement.

**Figure 4:**
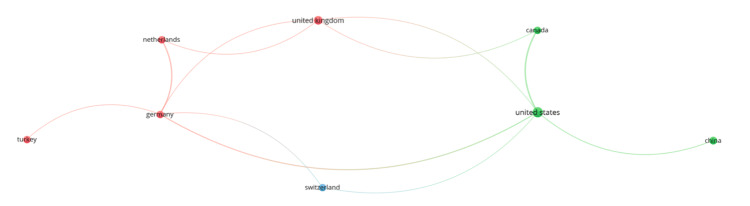
Country-level co-authorship network in iliofemoral DVT research DVT: deep vein thrombosis

Country-level analysis (Figure 4) identified the United States as the primary contributor, closely linked to Canada, Switzerland, and the United Kingdom. European participation was driven by Germany, the Netherlands, and Turkey, with Germany serving as a regional connector.

**Figure 5:**
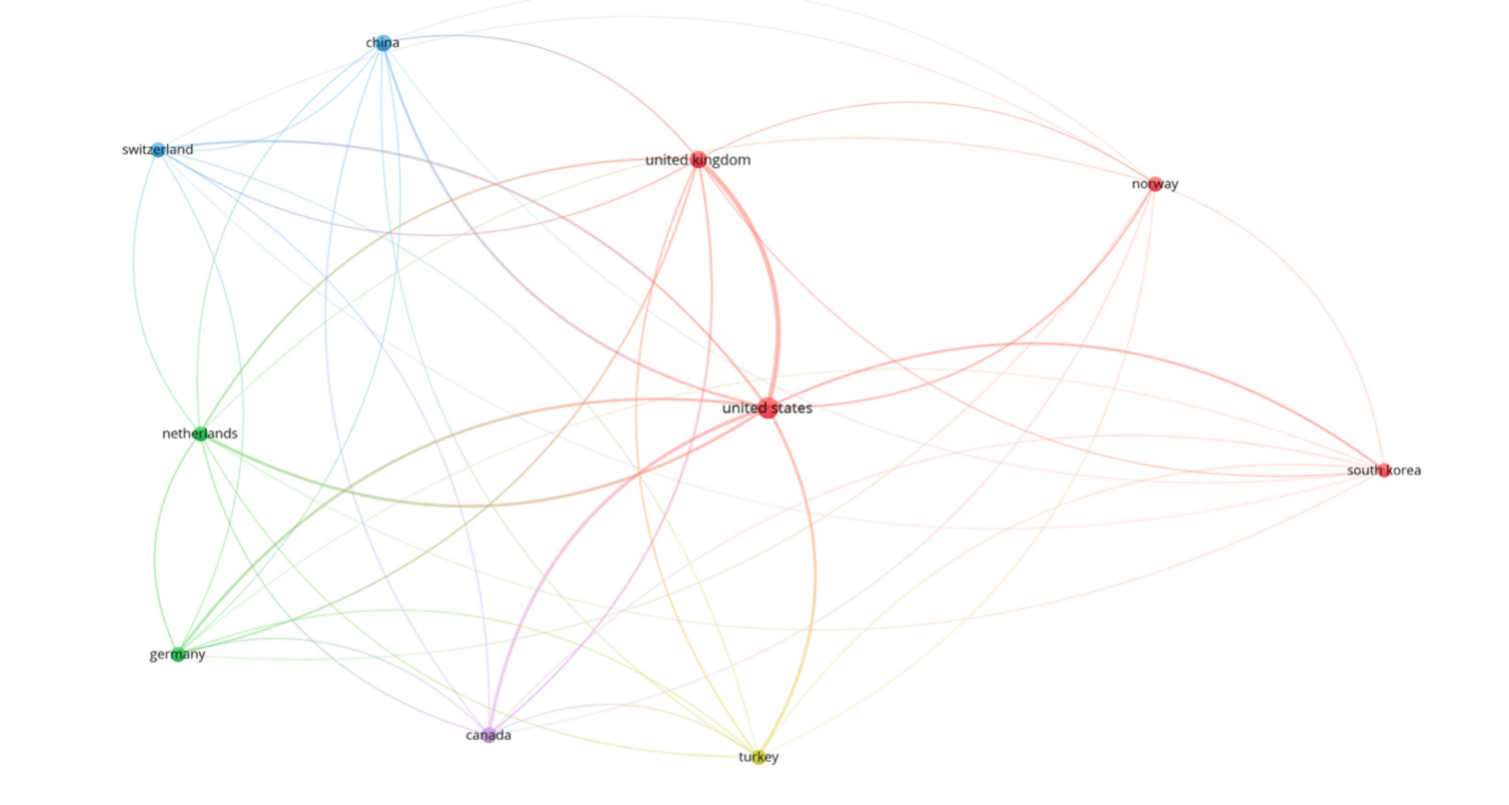
Expanded country-level co-authorship network in iliofemoral DVT research DVT: deep vein thrombosis

A more comprehensive international collaboration map (Figure 5) revealed a globally expanding network. Alongside dominant partnerships between North America and Europe, countries such as South Korea and China demonstrated increasing contributions and co-authorship connections. These findings suggest a maturing and increasingly integrated research field.

**Figure 6:**
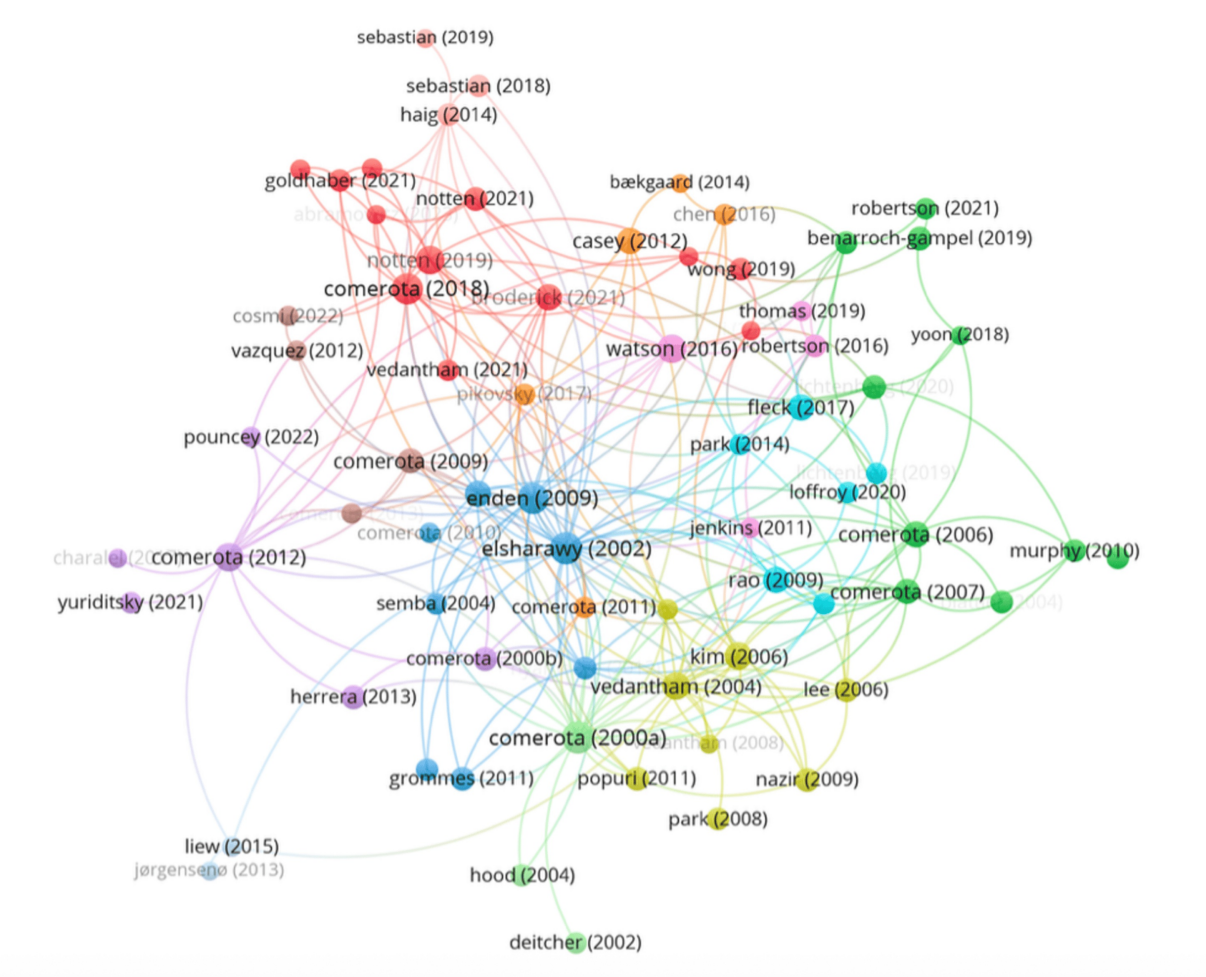
Co-citation network of frequently cited references in the iliofemoral DVT literature DVT: deep vein thrombosis

To assess the intellectual foundation of the field, a co-citation analysis was performed (Figure 6). The network revealed well-defined clusters representing distinct thematic cores, including clinical trial data, long-term outcome studies, and interventional techniques. Central nodes included works by Comerota, Vedantham, and Notten, underscoring their influence in shaping clinical discourse around thrombectomy in iliofemoral DVT. These references form the backbone of the citation landscape, frequently appearing across multiple publication clusters.

Discussion

This bibliometric analysis offers a comprehensive evaluation of global research trends in anticoagulation and mechanical thrombectomy for iliofemoral deep vein thrombosis (DVT) over a 25-year period. The notable increase in publication volume, particularly after 2020, reflects the growing interest in interventional treatments for proximal DVT, catalyzed by advancements in endovascular technologies and heightened clinical awareness of post-thrombotic syndrome (PTS) [1,2].

The shift in thematic focus from anticoagulant pharmacotherapy to mechanical thrombectomy corresponds with the evolving clinical evidence base. Seminal trials, such as ATTRACT and CAVENT, investigated the utility of catheter-directed thrombolysis and pharmacomechanical interventions, laying the foundation for clinical practice shifts and sustained academic engagement [3,4]. These studies, alongside subsequent registry-based evaluations, highlighted that while anticoagulation remains first-line therapy, selected patients with iliofemoral involvement may benefit from interventional approaches [8].

Citation patterns in this analysis reinforce the field’s intellectual structure. A small group of researchers has consistently contributed to multicenter studies, shaping the direction of research through high-impact clinical trials and reviews [2,3,9]. The co-citation clusters further emphasize the stability of this knowledge base.

Geographically, research output has been dominated by North American and European countries, particularly the United States, the United Kingdom, and Germany. The prominence of U.S.-based institutions, such as Washington University and Harvard University, aligns with the country's leadership in sponsoring and executing large-scale vascular trials [9,10]. However, the increasing participation of institutions from China, Turkey, and South Korea signals a welcome trend toward global inclusion and collaboration in thrombosis research [11].

The collaboration maps highlight the importance of sustained research partnerships. Strong co-authorship networks, particularly those formed through major trials such as ATTRACT, illustrate how long-term collaboration contributes to both academic influence and clinical impact [3,12].

Strengths and limitations

A major strength of this study lies in its use of Dimensions AI, which offers wide bibliometric coverage and integrates citation tracking, research categories, and author affiliations. The combined use of co-authorship, institutional, and co-citation network analyses enables a multidimensional understanding of productivity and influence in the field.

However, this study has limitations. The inclusion of only English-language peer-reviewed articles may have excluded relevant work published in other languages or within gray literature. Additionally, although Dimensions provides broad indexing, some important metrics available in databases such as Scopus or Web of Science (e.g., h-index variations, funding metadata) were not captured.

Future directions

While anticoagulation remains the foundation of DVT management, mechanical thrombectomy is increasingly supported by device innovation and growing observational data. Newer technologies, such as the ClotTriever system, offer promising results without the need for thrombolytics, potentially reducing bleeding risk in high-risk patients [13]. However, several gaps identified in this bibliometric analysis warrant further attention.

First, there is a noticeable lack of randomized controlled trials evaluating the effectiveness of new-generation thrombectomy devices across ethnically diverse populations. Most existing evidence stems from North American or European cohorts, limiting generalizability to other regions.

Second, the analysis revealed minimal representation from Global South countries, particularly in Africa, Southeast Asia, and parts of Latin America, in both authorship and institutional collaboration networks. This underrepresentation highlights the need for more inclusive international research partnerships that can capture region-specific disease patterns, health system contexts, and patient outcomes.

Third, the bibliometric findings underscore the absence of widely adopted, standardized outcome metrics in thrombectomy research. Heterogeneity in reported endpoints, such as thrombus removal efficiency, symptom resolution timelines, and quality of life measures, complicates comparison across studies and hinders the development of evidence-based guidelines.

Future research should prioritize collaborative multicenter trials, particularly in underrepresented settings, and work toward consensus on outcome definitions to enable clearer comparisons of therapeutic efficacy.

## Conclusions

This bibliometric analysis provides a comprehensive overview of global research trends related to anticoagulation and mechanical thrombectomy for iliofemoral deep vein thrombosis (DVT) over the past 25 years. The findings demonstrate a significant increase in scholarly output, particularly in the last decade, with a growing emphasis on interventional approaches alongside traditional pharmacologic management. The analysis identified a small group of influential authors and institutions that have shaped the field through high-impact studies and sustained collaboration. The United States emerged as the most productive and collaborative country, with increasing contributions from Europe and Asia.

Co-authorship and co-citation network visualizations revealed strong scientific partnerships and a consolidated knowledge base anchored by landmark clinical trials. These insights not only highlight the maturity of the field but also underscore the need for expanded international collaboration and future research that leverages real-world evidence and multicenter data. This study serves as a foundation for guiding future research priorities and fostering global academic engagement in the evolving management of iliofemoral DVT.
